# Feasibility of controlling CD38-CAR T cell activity with a Tet-on inducible CAR design

**DOI:** 10.1371/journal.pone.0197349

**Published:** 2018-05-30

**Authors:** Esther Drent, Renée Poels, Manon J. Mulders, Niels W. C. J. van de Donk, Maria Themeli, Henk M. Lokhorst, Tuna Mutis

**Affiliations:** Department of Hematology, VU University Medical Center, De Boelelaan, Amsterdam, The Netherlands; Mie University Graduate School of Medicine, JAPAN

## Abstract

Recent clinical advances with chimeric antigen receptor (CAR) T cells have led to the accelerated clinical approval of CD19-CARs to treat acute lymphoblastic leukemia. The CAR T cell therapy is nevertheless associated with toxicities, especially if the CARs are not entirely tumor-specific. Therefore, strategies for controlling the CAR T cell activity are required to improve their safety profile. Here, by using the multiple myeloma (MM)-associated CD38 molecule as target molecule, we tested the feasibility and utility of a doxycycline (DOX) inducible Tet-on CD38-CAR design to control the off-target toxicities of CAR T cells. Using CARs with high affinity to CD38, we demonstrate that this strategy allows the proper induction of CD38-CARs and CAR-mediated T cell cytotoxicity in a DOX-dose dependent manner. Especially when the DOX dose was limited to 10ng/ml, its removal resulted in a relatively rapid decay of CAR- related off-tumor effects within 24 hours, indicating the active controllability of undesired CAR activity. This Tet-on CAR design also allowed us to induce the maximal anti-MM cytotoxic activity of affinity-optimized CD38-CAR T cells, which already display a low toxicity profile, hereby adding a second level of safety to these cells. Collectively, these results indicate the possibility to utilize this DOX inducible CAR-design to actively regulate the CAR-mediated activities of therapeutic T cells. We therefore conclude that the Tet-on system may be more advantageous above suicide-genes to control the potential toxicities of CAR T cells without the need to destroy them permanently.

## Introduction

Over the past years, the clinical successes of chimeric antigen receptor (CAR) engineered T (CAR T) cells have evoked a tremendous enthusiasm for this new mode of immunotherapy in the battle against cancer [1–7]. On the other hand, the increasing clinical experience with CAR T cell therapy have made the investigators aware of the possible severe, even fatal toxicities of this powerful approach [4,8–10]. Several currently known toxicities of CAR T cells are associated with their *in vivo* uncontrolled growth and excess cytokine release soon after infusion in the patients, most probably—though not entirely- due to the on-target off-tumor activities of CAR T cells. This is considered an important concern since virtually all CAR T cells developed to date, including the most successful CD19 CAR T cells, are directed against tumor-associated, but not entirely tumor specific antigens [11,12].

Set out to develop an efficient CAR T cell therapy for multiple myeloma (MM) we also have recently investigated and demonstrated the possibility to target MM cells with CAR T cells directed against the CD38 antigen, which is highly and uniformly expressed on MM cells [13]. Although CD38 is also expressed on normal hematopoietic cells at intermediate levels, we have shown that CD38-CAR T therapy can be very selective for MM cells, using affinity-optimized CD38-CAR T cells [14]. However, if the affinity of the CAR is not carefully optimized, high affinity CD38-CARs, like many others, can readily cause on-target, off-tumor side effects [8,15,16]. Therefore, an active *in vivo* control of CAR T cell activity is also highly desirable for a safer CAR T cell therapy. Towards this goal, the most frequently proposed and applied strategy is to equip therapeutic T cells with the so-called suicide genes. For instance the herpes simplex virus thymidine kinase (HSV-TK), which converts the prodrug ganciclovir (GCV) into a toxic product [17], or the inducible caspase9, which is dimerized by a small molecule to induce apoptosis [18–20]. The therapeutic T cells can also be engineered to aberrantly express surface antigens like CD20 or EGFR [21,22], which enables their specific targeting via antibodies.

While the suicide gene approach had been proven effective in experimental and in the clinical settings, it may not be the ideal strategy to control CAR T cells, since once these genes are activated, the therapeutic effect is also lost permanently. Therefore, actively controlling the CAR expression at the cell surface, rather than killing the CAR expressing T cells, may provide better opportunities to improve their safety profile. Aiming at this goal, several innovative strategies have already been proposed, such as the inducible dimerization of the intra and extracellular domains of the CAR[23–25], or a CTLA-4 signaling-mediated shuttling to the cell membrane [26] (different approaches reviewed in [27] and [28]). While such novel strategies are in full development, a traditional way of controlling the transgene expression is through a tetracycline or doxycycline (DOX) inducible on- (Tet-on) or off- (Tet-off) switch. Indeed, this strategy has recently been successfully applied for controlling the CD19-CAR expression [29].

Due to its relative convenience, we here investigated the utility of the Tet-on inducible CAR design to effectively and timely control the cytotoxic activity of CD38-CAR T cells. Our results demonstrate that the Tet-on CAR design can indeed control the expression of even high affinity CD38-CARs to effectively allow their CD38-dependent cytotoxic activity in a DOX-dose-dependent manner. Using a carefully defined dose of DOX the CD38-CAR expression and thereby all associated effector functions decayed rapidly upon DOX removal, whereby minimizing the undesired off-tumor effects. Our results thus indicate the feasibility of actively and timely controlling the CD38-CAR T cell activity in case of undesired toxicity associated with on-target, off-tumor effects.

## Material and methods

### Retroviral vector construction

The Tet-on 3G inducible system (Clontech) consists of, the pRetroX-TRE3G vector with the *P*_TRE3GV_ inducible promoter and the Mock or CAR together with the pRetroX-TET3G for the transactivator protein. The high affinity CD38-CAR028, (*K*_D_ = 1.8 nM, binding kinetics on CD38^+^ cell line: EC_50_ = 0.3 ng/ml) [13,14] and low affinity CD38-CARB1 (*K*_D_ = not applicable. EC_50_ = 4.3 ng/ml) and CARA4 (*K*_D_ = 1915 nM. EC_50_ = 3.3 ng/ml) genes [14] were amplified with primers containing the SgrAI and ClaI restriction sites, (forward 5’GGTCCAATCGATATGGCGCTGCCTGTGAGCTC -3’, reverse 5’- CGTTACTAGTGGA CACCGG CGTCCTCATCTAG -3’). PCR products were purified using gel-clean up (Bioké) and were subsequently ligated into the pRetroX-TRE3G vector with a T4 ligase (Sigma).

### Generation of retroviral particles and transduction of T cells

GP2 293 packaging cells (Clontech) were calcium phosphate transfected with 10 μg pRetroX-TRE-CAR or Mock and pRetroX-TET3G constructs + 5 μg gag-pol (pHIT60) (Roche), and 5 μg envelope (pAmpho) vectors (Clontech). 16 hours post-transfection complete medium (DMEM + 10% FBS (Clontech)) was refreshed, and two and three days after transfection, cell free supernatants containing retroviral particles were collected and directly used for transduction.

Peripheral blood mononuclear cells (PBMCs) from healthy donors were stimulated with lectin-like phytohemagglutinin (PHA-L) (Sigma) in a 6 well plate in culture medium (RPMI-1640, 10% Tet-approved FBS, penicillin; 10.000 U/ml, streptomycin; 10,000 μg/ml). After 48 hours, cells were transferred to retronectin (Takara) coated 6-well plates (Falcon). Retroviral transduction was performed by addition of 1 ml TRE-CAR/Mock virus + 1 ml TET virus per well followed by spinoculation (3000 rpm, 1 hour at room temperature) in the presence of 4 μg/ml Polybrene. A second transduction was conducted after 16 hours. 6–8 hours after the second hit, half of the cell supernatant was replaced by fresh culture RPMI-1640 + 10% tet-approved FBS + 50 IE/ml rhIL-2 (Proleukin®, Novartis).

### Transduced T cell selection and expansion

3 x 10^6^ T cells were selected with neomycin (80 ug/ml) for 1 week and puromycin (5 ng/ml) for 3 days after transduction. Based on the fraction of surviving cells after neomycin and puromycin selection, the initial double transduction efficiency was estimated to be around 15–30%). ~0,5–1 x 10^6^ selected T cells were expanded in RPMI-1640 (Invitrogen) + 10% Tet-approved FBS (Clontech) + antibiotics (penicillin; 100 U/ml, streptomycin; 100 μg/ml) using a feeder cell/cytokine mixture consisting of irradiated EBV cell lines of 2 donors (50 Gy) and allogeneic PBMCs of 3 donors (25 Gy),100 U/ml IL-2 and 1 ng/ml PHA-L.

### Cell lines

Unmodified or luciferase (Luc-GFP)-transduced human MM cell lines, UM9 [30] and RPMI8226 [31] were cultured in RPMI-1640 (Invitrogen) + 10% FBS (Invitrogen) + antibiotics (penicillin;100 U/ml, streptomycin; 100 μg/ml) as described [13,14].

### Primary cells from MM patients and healthy individuals

Bone marrow mononuclear cells (BM-MNC) containing ~20% malignant plasma cells were isolated from bone marrow aspirates of MM patients through Ficoll-Paque density centrifugation and either used directly or cryopreserved in liquid nitrogen until use. PBMCs/MNCs were isolated from Buffy coats of healthy blood-bank donors by Ficoll-Paque density centrifugation.

### Production of soluble CD38 extracellular (sCD38) domain and CAR staining with sCD38

Cloning, expression and purification of recombinant CD38 protein was executed as previously described [14]. In brief, cell were washed twice in PBS + 4% human serum albumin, followed by the first staining with sCD38 (30 minutes), washed twice and stained with a PE-conjugated anti-His antibody (Biolegend) for 15 minutes.

### Flow cytometry

Flow cytometry assays were performed on BD LSRFortessa. Viable cells were determined with live/dead cell marker (LIVE/DEAD® Fixable Near-IR; Life Technologies L10119). Transduction efficiency and associated CAR expression was measured with an monoclonal antibody towards NGFR-APC (CD271) (clone ME20.4 Biolegend). Monoclonal antibodies used for cytotoxicity assays: CD3-Fitc (clone SK7), CD14-PerCP (clone MoP9), CD19-PerCP (clone SJ25C1) and CD38-PE (clone HB7) (BD Bioscience). CD56-PC7 (clone N901) and CD138-APC (clone BA38) (Beckman Coulter). To distinguish Mock/CAR T cells from target cells, target cell were stained with 0.5 μM Violet tracer (Thermo Fisher C34571) for 25 minutes and washed before cytotoxicity assay co-cultures. Flow cytometry data analysis was performed with FACS Diva 6.1 software.

### Cytokine measurements

To determine cytokine production by TRE-Mock and TRE-CAR T cells, cell supernatants were harvested 24 hours after co-culture with MM-BM at an E:T ratio of 3:1. The cytokine content of the supernatants was measured by Cytokine Bead Array (CBA) Human Th1/Th2/Th17 cytokine kit (BD) according to manufacturer’s protocol. Beads were washed and analyzed by a standardized flow cytometry assay.

### Bioluminescent and flow cytometry-based cytotoxicity assays

One to three days after transduction, selection and expansion, inducible CD38-CAR T cells were incubated with Luc-GFP-transduced human malignant cell lines or violet tracer (Thermo Fisher) labeled primary BM-MNC for 24 hours. The luciferase signal produced by surviving malignant cell lines was determined after 24 hours with a GloMax® 96 Microplate Luminometer (Promega) within 15 minutes after the addition of 125 μg/mL beetle luciferin (Promega). % lysis cells = (1 − (BLI signal in treated wells / BLI signal in untreated wells)) × 100%. To analyze surviving primary BM-MNCs Flow-Count™ Fluorospheres (Beckman 7547053) were added, cells were harvested and stained for different CD markers (see above). Viable cells were then quantitatively analyzed through Flow-Count-equalized measurements. Percentage cell lysis was calculated as % lysis cells = (1 − (absolute number of viable target cells in treated wells / absolute number of viable target cells in untreated wells)) × 100%.

### Ethical statement

Bone marrow samples from MM patients and peripheral blood from healthy controls (all > = 18 years of age) were taken after written informed consent in accordance with the declaration of Helsinki. Whenever necessary, the study design, including blood/ bone marrow sampling procedures was submitted to and approved by the VU university medical ethical committee, Amsterdam.

### Statistical analysis

Statistical analyses were performed using Graphpad Prism software version 7.0. For normal distributions parametric student’s t-tests were used. In analyses where multiple groups were compared, either a parametric ANOVA with bonferroni posthoc test or nonparametric Kruskal-Wallis test were used with subsequent multiple comparison. A p value <0.05 was considered significant.

## Results

### DOX dependent induction of CD38-CAR expression

**Fig 1:**
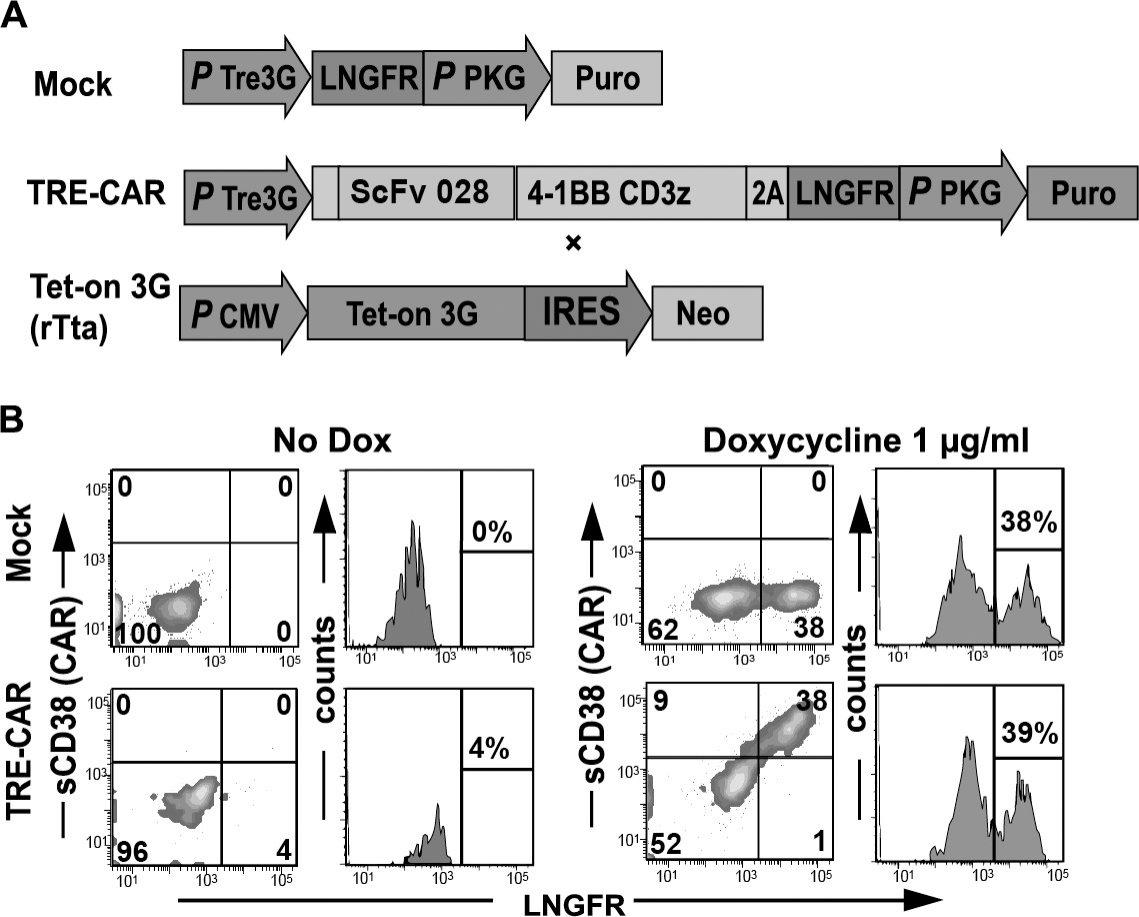
DOX induced CD38-CAR expression. (A) Schematic overview of constructs. The pRetroX-TRE3G vector with the P_TRE3GV_ inducible promoter controlling the transcription of Mock containing the marker LNGFR or the CD38-CAR (shown is high affinity scFv 028; the same vector design is also used for low affinity CARs A4 and B1), consisting of the single chain variable fragments, 4-1BB and CD3ζ and a LNGFR separated by a P2A sequence. These vectors were co-transduced with the pRetroX-TET-On 3G containing the transcription site for the transactivator protein rTta. (B) Representative flow cytometry density plots and histograms to determine CAR expression of the inducible CAR T cells, after 48 hours incubation with 0 or 1000 ng/ml DOX. The expression of the marker LNGFR was measured with an APC-conjugated antibody. CAR expression was measured by binding of his-tagged (HHHHHH) soluble CD38 (sCD38) protein to the ScFv domain, stained with PE-conjugated anti-His tag antibody.

To evaluate the controllability of CAR expression with an inducible design, we first generated a Tet-on inducible second generation CD38-CAR, which contained single chain variable fragments (scFv) with a high CD38 affinity, including the 4-1BB and CD3ζ signaling domains. This CAR gene was put under the regulation of a third generation pTre, containing seven tetracycline responses elements (TRE) followed by a minimal CMV promotor (Fig 1). The construct also contained the low affinity nerve growth factor receptor (LNGFR), separated from the CAR gene by a P2A sequence. The control mock vector contained only the LNGFR marker gene (Fig 1A). Upon retroviral transduction with this inducible construct (TRE-CD38-CARs), the T cells showed no detectable CAR expression in the absence of DOX (Fig 1A) but expressed high levels of the CAR within 48 hours of exposure to a high dose[32] of DOX (Fig 1B). All transduced cells, including the mock-transduced cells also expressed the LNGFR marker gene (Fig 1B).

**Fig 2:**
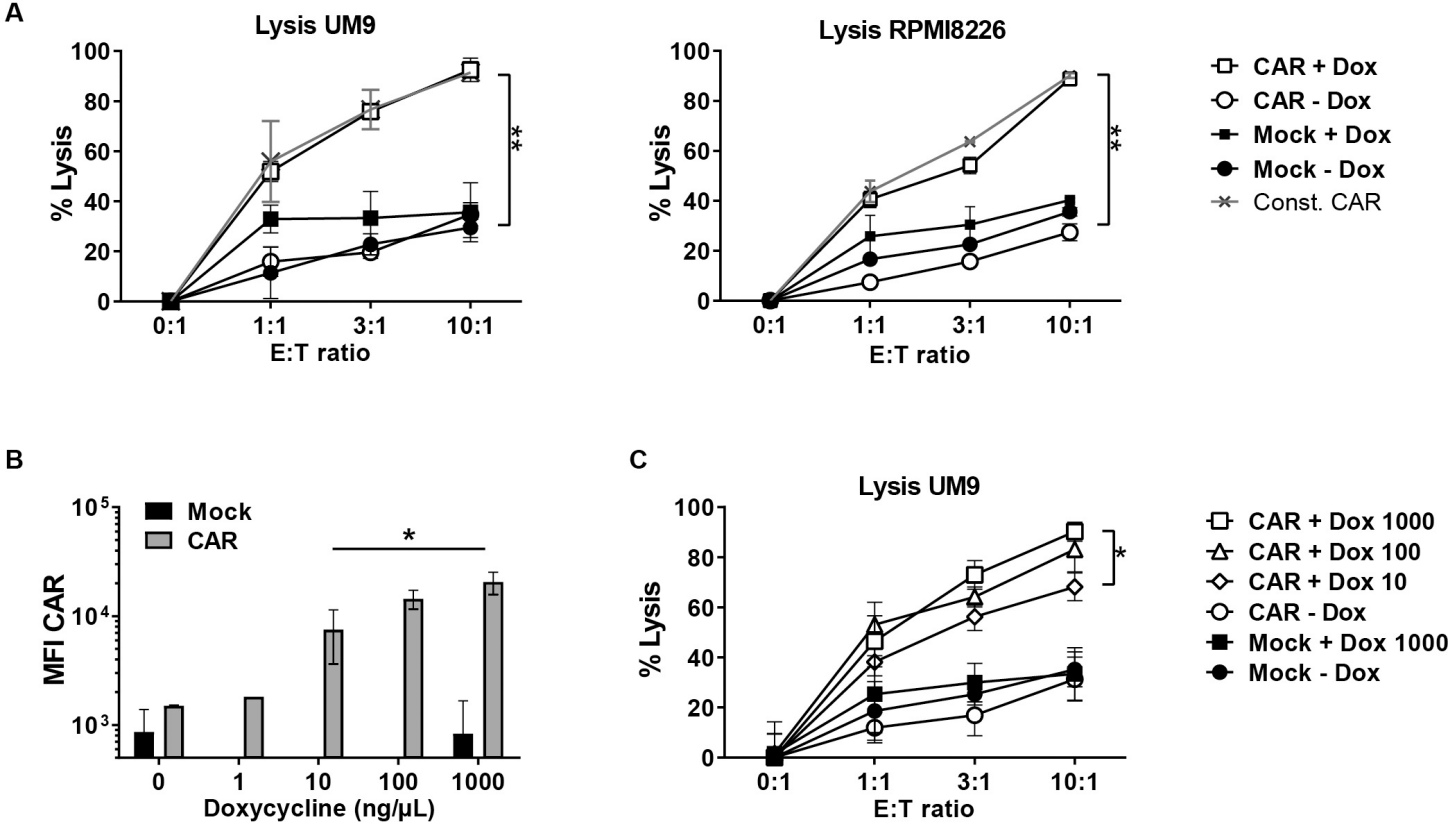
DOX dose-dependent induction of CD38-CAR expression and anti-MM cytotoxicity. (A) Lysis of luciferase-transduced CD38^+^ MM cell lines UM9 and RPMI8226 after co-incubation with Mock and inducible, high affinity (028) CD38-CAR, after treatment with no or 1000 ng/ml DOX for 48 hours. Grey lines indicate the lysis by constitutively expressed high affinity (028) CD38-CAR T cells The BLI signal from surviving MM cells was measured after 16 hours using a luminometer and the percentage lysis was calculated as indicated in the material & methods. Presented is the pooled data from 2 independent experiments. Error bars indicate the mean +/- SD (B) Mean fluorescent intensity (MFI) of the CAR measured by staining with soluble CD38-his after 48 hours incubation with 0, 1, 10, 100 or 1000 ng/ml DOX, Presented is the pooled data from 2 independent experiments. Error bars indicate the mean +/- SD. (C) The cytotoxic activity of untreated or DOX treated inducible CD38-CAR T cells against luc+ MM cell line UM9 after 16 hours. Presented is the pooled data from 2 independent experiments. Error bars indicate the mean +/- SD. In all panels * indicates p value <0.05 and ** <0.01 using one-way analysis of variance and subsequent multiple comparison.

We then determined the cytotoxic activity of T cells transduced with this inducible CD38-CAR against two CD38^+^ MM cell lines UM9 and RPMI8226 with or without pre-treatment with a high concentration (1000ng/ml) of DOX (Fig 2A). We also compared the results with those obtained from T cells that constitutively expressed the high affinity 028 CD38-CAR. As expected, there was no CAR expression and no CAR-mediated lysis in the absence of DOX. In contrast, DOX- treatment induced CAR expression and resulted in the effective lysis of both MM cell lines within 16 hours (Fig 2A) at similar levels observed from constitutive CAR expressing T cells, indicating the efficacy of the inducible CAR design. The lysis levels further increased during longer (>120 hours) co-incubations (S1A Fig). Furthermore, it appeared possible to re-induce functional CAR expression with DOX after an initial DOX withdrawal (S1B Fig).

### DOX dose-dependency of CD38-CAR expression and cytotoxic activity

We next evaluated whether the level of CD38-CAR expression and the CD38-dependent cytotoxic activity of TRE-CD38-CAR T cells could be regulated by the dose of DOX, by incubation with serial concentrations of DOX ranging from 1–1000 ng/ml for 48 hours (Fig 2B)[32,33]. The CAR expression was maximal at 1000 ng/ml of DOX, but gradually decreased by lowering the dose, which was also reflected in the cytokine production (S2 Fig). A 5-fold lower expression level was reached at a DOX dose of 10 ng/ml (Fig 2B). The cells showed a very low CAR expression at a dose 1 ng/ml, which was not distinguishable from DOX untreated conditions. The cytotoxic activity of the T cells also significantly and proportionally decreased by lowering the dose of DOX, from 95% of lysis at 1000 ng/ml to 63% of lysis at 10 ng/ml DOX at an effector: target ratio of 10:1 (Fig 2C). Again here, no CD38-mediated cytotoxic activity above non-specific (mock) levels was observed from DOX untreated TRE-CD38-CAR T cells, indicating that there was no functional “leakage” in this inducible system, despite the fact that a very low level of CD38-CAR was detectable on the cell surface (Fig 2B).

### Decay kinetics of CAR expression and CD38-dependent on-tumor cytotoxic activity of TRE-CD38-CAR T cells after DOX removal

**Fig 3:**
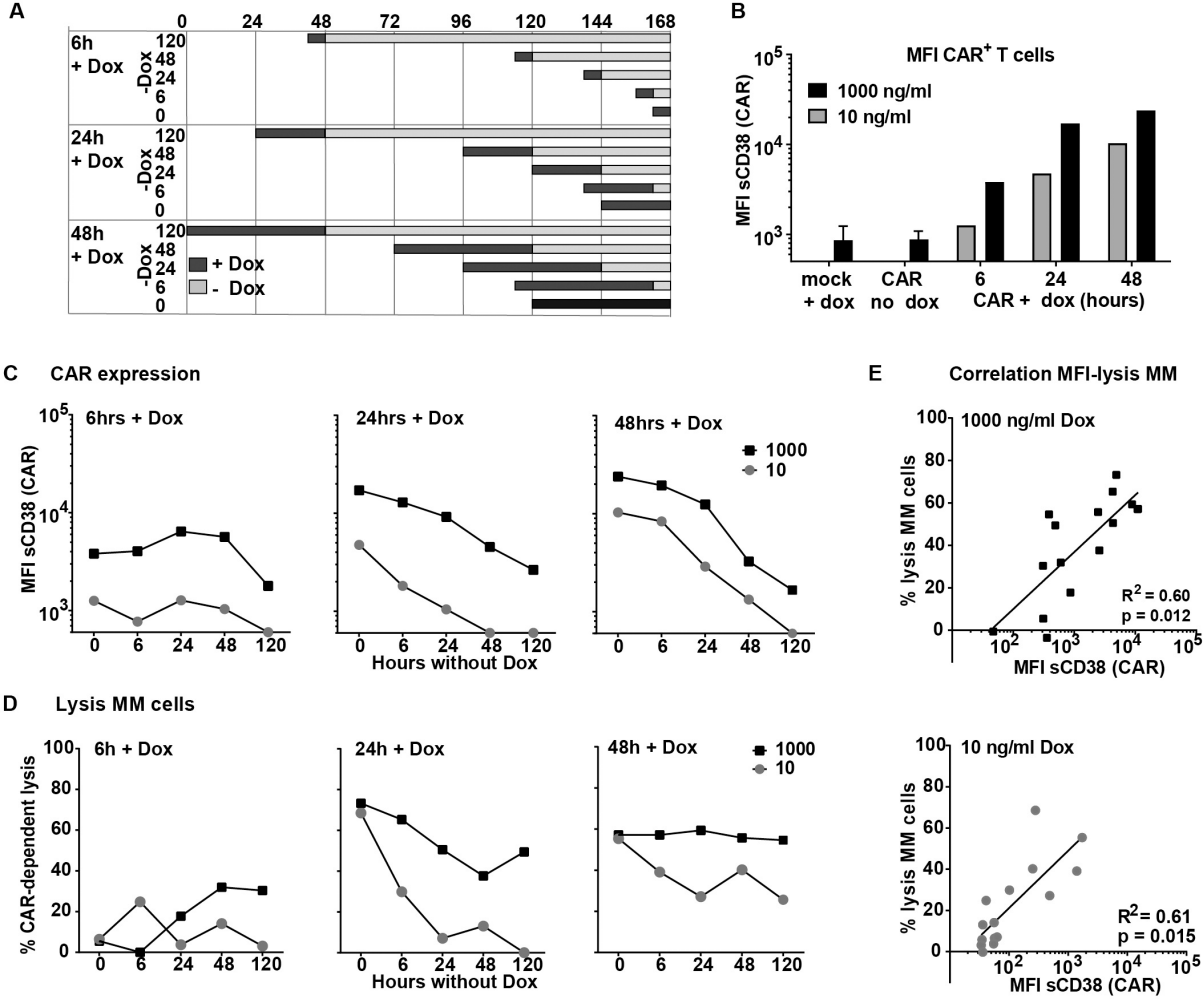
Induction and decay kinetics of CD38-CAR expression. (A) schematic overview of CAR induction and decay assay. Black bars indicate the DOX incubation times, gray bars indicate the period of decay after the removal of DOX. (B and C) Representative results of five independent experiments of mean fluorescent intensity (MFI) of the CAR measured by staining with soluble his-tagged CD38 after 6, 24 or 48 hours incubation with (B) 10 or 1000 ng/ml DOX or 6, 24, 48 or 120 hours after washing of DOX (C) (an MFI of 600, observed by Mock cells was considered background expression). (D) A MM patient bone marrow sample with 20% MM cells was co-incubated with inducible, high affinity (028), CD38-CAR T cells (E:T ratio 3:1) treated with DOX according to the schedule depicted in Fig 3A. are the CAR-dependent % lysis of CD138+/CD38+ MM cells (% lysed by CAR—% lysed by Mock). Presented is the representative data of n = 5. (E) Significant Pearson correlation of MFI of CAR expression as detected with soluble CD38 (sCD38) with % lysis of MM cells. High dose DOX R^2^ = 0.60 and p = 0.012, low dose DOX R^2^ = 0.61 and p = 0.015.

After showing the DOX dose-dependency of CAR expression, we studied the induction and decay kinetics of the CD38-CARs upon exposure and after the withdrawal of serial concentrations of DOX (S3 Fig). We also evaluated the CD38-dependent cytotoxic activity of the cells against MM cells in these assays. The experimental set-up is depicted in Fig 3A and time and concentration-dependent CAR expression levels in Fig 3B. Six hours of DOX treatment induced a slight to moderate CAR expression, even with 1000 ng/ml of DOX. This moderate expression did not translate immediately into a specific lysis but CAR expression retained until 48 hours after DOX removal (Fig 3C left panel). Remarkably, after DOX removal, we observed a maximal 30% CD38-CAR-mediated lysis of MM cells above the mock control at 48 and 120 hours (Fig 3D left panel), possibly due to a relatively slow rate of transcription induced by short term DOX incubation.

A 24-hour DOX-treatment resulted in substantial CAR expression (Fig 3C middle panel) and effective CD38-CAR mediated lysis of MM cells (Fig 3D middle panel). The CAR decay upon DOX removal occurred in a linear fashion, with a slightly faster decay kinetics for the cells incubated with 10 ng/ml of DOX as compared to 1000 ng/ml. The cells expressed very low but detectable levels of CARs 24 hours after DOX removal, but no CAR expression was detectable 48 hours after DOX removal (Fig 3C middle panel). The cytotoxic activity of the cells decreased in a significantly faster kinetics after 10 ng/ml DOX incubation. No significant CAR mediated lysis of MM cells above mock control could be observed 24h after of removal of 10 ng/ml DOX (Fig 3D, middle panel).

As expected, the CAR expression was highest after 48-hour DOX treatment. The CAR decay kinetics was similar to 24-hour incubated cells but the cells still expressed intermediate to moderate levels of CAR for longer periods, since the initial levels were higher (Fig 3C right panel). Consequently, the cells treated with 1000 ng/ml DOX did not significantly downregulate their high anti-MM activity, while a 30–40% CAR mediated cytotoxic activity against MM cells remained even 48–120 hours after the removal of 10 ng/ml DOX (Fig 3D right panel). When we correlated the CAR expression levels and the lysis levels in these assays, we observed a correlation between CAR expression and CAR-mediated lysis of the MM cells for both 1000 (Fig 3E top panel) and 10 ng/ml of DOX (Fig 3E lower panel).

### Decay kinetics of CD38-dependent off-tumor cytotoxic activity of TRE-CD38-CAR T cells after DOX removal

**Fig 4:**
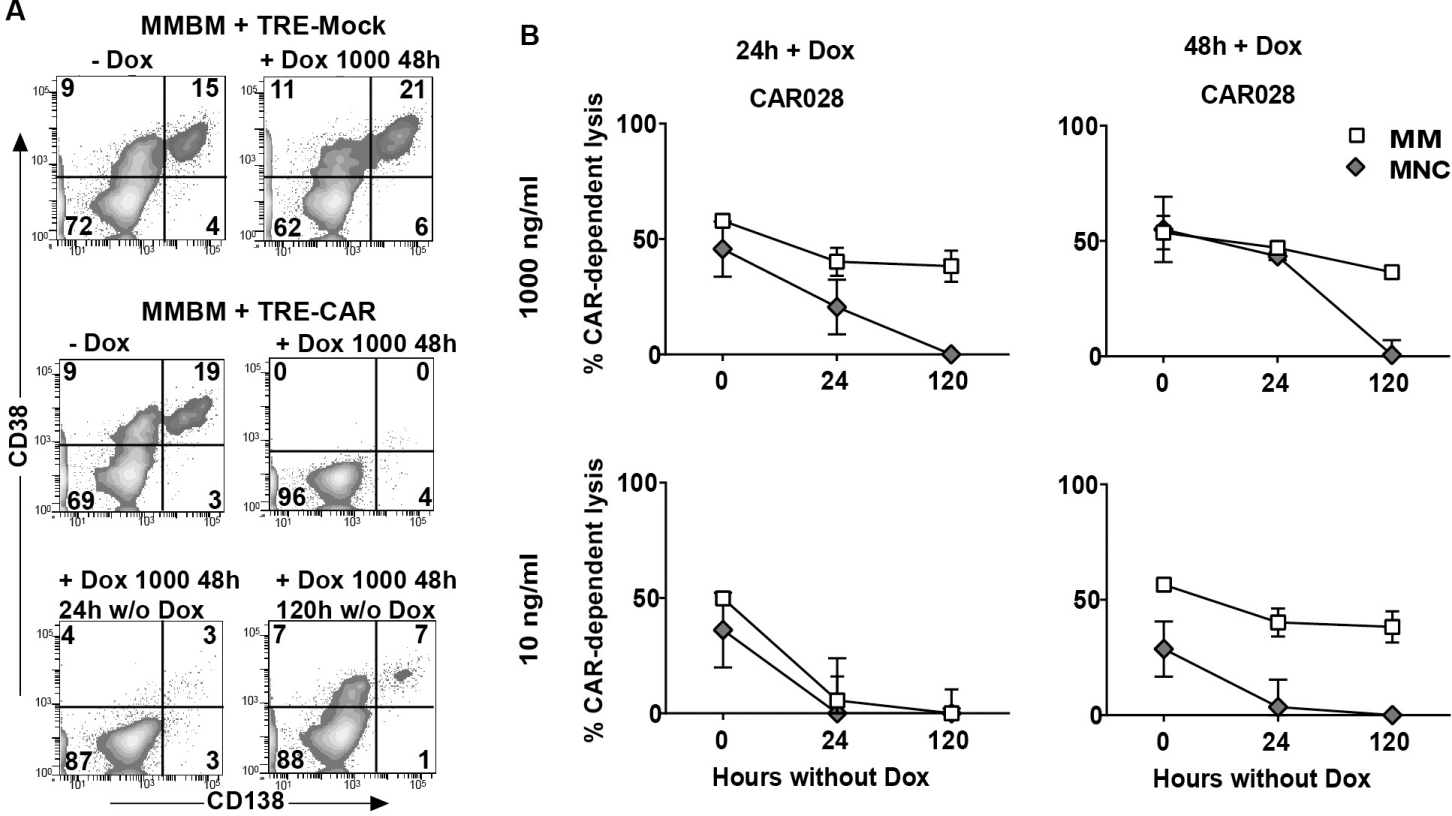
Off-tumor effect of inducible CD38-CAR T cells. (A) Representative flow cytometry density plots of MM-BM with CD38^+^/CD138^+^ cells (MM) after treatment with inducible mock (+/- 1000 ng/ml DOX for 48 hours) and inducible high affinity (028) CD38-CAR T cells (- DOX or + 1000 ng/ml DOX for 48 hours and 0, 24 or 120 hours after DOX removal). (B) Pooled data obtained from the analysis of five MM patient bone marrow samples (patient 1–5, see for their phenotype data S4 Fig) with ~20% MM cells (S4 Fig) were co-incubated with inducible high affinity (028) CD38-CAR T cells (E:T ratio 3:1) treated with DOX according to the schedule Fig 3A. Shown are the mean CAR-dependent % lysis of MM (CD138^+^/CD38^+^ ; open squares) and % lysis of healthy non-MM cells (CD138^-^/CD56^-^/CD38^+/-^; grey diamonds) by inducible CD38-CAR T cells. Incubated with DOX for 24 hours 1000 ng/ml (upper left), 48 hours 1000 ng/ml (upper right), 24 hours 10 ng/ml (lower left), 48 hours 10 ng/ml (lower right). Presented is the pooled data from 5 independent experiments. Error bars indicate the mean +/- SEM. (Pt 1–5, S4 Fig).

An important aim of the inducible CAR-design is to effectively and rapidly control the on-target off-tumor mediated toxicities of high affinity (028) CD38-CAR T cells. Therefore, in the further evaluation of TRE-CD38-CAR T cells we not only tested their anti-MM activity but also evaluated the potential off-tumor toxicities against CD38^int^ normal hematopoietic cells upon exposure and after removal of DOX. To test this in a most relevant way, we used BM samples from MM patients, which contain not only CD38^hi^ MM cells but also CD38^int^ normal hematopoietic cells as target cell populations. After the induction of CAR expression the TRE-CD38-CAR T cells were incubated with BM-MNC and the CAR-dependent lysis of various cell subsets was determined by flow cytometry-mediated assays as described previously [13,14]. An illustrative example of such an assay is depicted in Fig 4A. As depicted in Fig 4B, both 24h and 48h stimulation with 10 or 1000 ng/ml DOX of TRE-CD38-CAR T cells resulted in substantial CAR-mediated of MM cells. As expected from the high affinity (028) CD38-CAR T cells there was also considerable lysis of CD38+ non-MM cells in the bone marrow. After the removal of DOX, however, the TRE-CD38-CAR T cells which were treated with 10 ng/ml DOX rapidly lost their off-tumor effects within 24 hours, while there was still 50%-MM activity left especially of CAR T cells that were exposed to DOX for 48 hours. 120 hour after DOX removal only the cells that were exposed to 1000 ng/ml DOX retained some anti–MM activity; all off-tumor activity was lost. These results indicated that the off-tumor activities of high-affinity CD38-CAR T cells can be readily and rapidly down-regulated after exposure of the cells even 48 hours to relatively low doses of (10ng/ml) DOX, while some anti-MM reactivity still retained.

### Low-affinity inducible CD38-CAR T cells

**Fig 5:**
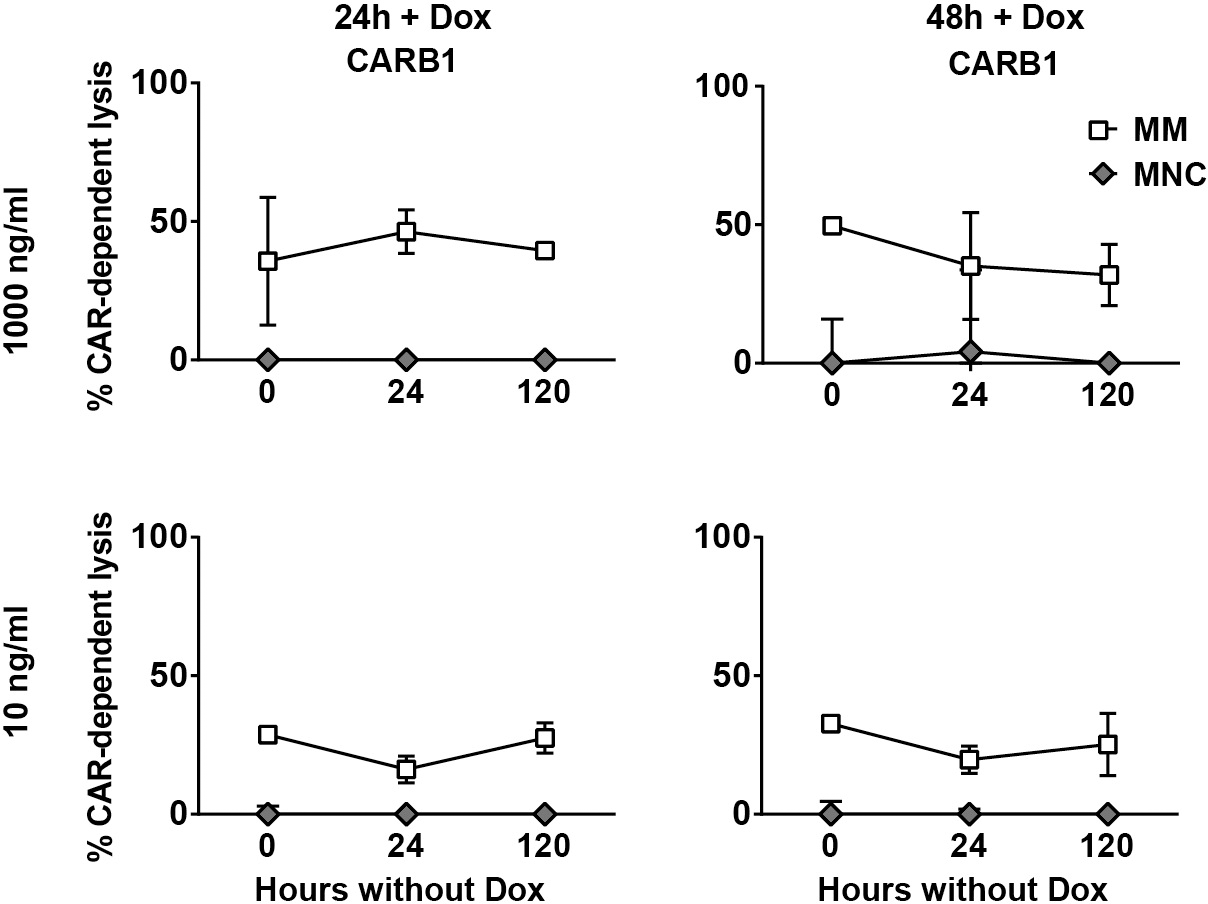
Off-tumor effect of inducible low affinity CD38-CAR T cells. MM patient bone marrow samples (n = 4) with ~20% MM cells were co-incubated with inducible low affinity (B1) CD38-CAR T cells (E:T ratio 3:1) treated with DOX according to the schedule Fig 3A. Depicted are the average CAR-dependent lysis of MM cells (CD138^+^/CD38^+^ ; open squares) and lysis of healthy non-MM cells (CD138^-^/CD56^-^/CD38^+/-^ ; grey diamonds) by inducible CD38-CAR T cells. Incubated with DOX for 24 hours 1000 ng/ml (upper left), 48 hours 1000 ng/ml (upper right), 24 hours 10 ng/ml (lower left), 48 hours 10 ng/ml (lower right). Presented is the pooled data from 4 independent experiments. Error bars indicate the mean +/- SEM. (Pt 2–5, same pts as in Fig 4 and S4 Fig).

The experiments addressing the on- and off-tumor effects of inducible high affinity (028) CD38-CAR T cells indicated that gradual decay of CAR expression after DOX removal not only allows the rapid and effective control of the off-tumor toxic effects, but may also generate a small, albeit a temporary therapeutic window in which the anti-tumor effects can be maintained. Thus not only optimally lowering the affinity of CD38-CARs, as we have recently shown [14] but also lowering the CAR expression on the cell surface seemed to result in discrimination of CD38^high^ MM cells from CD38^int^ normal hematopoietic cells. Therefore we finally questioned whether these two strategies can be combined to make much safer CAR T cells. Hence we generated inducible CD38-CAR T cells from (two) a low affinity CARs (CAR A4 and B1), which displayed much less off-tumor toxicity profiles than the high affinity CD38-CAR T cells[14]. After induction of CAR expression with 10 or 1000 ng/ml DOX the low affinity CAR T cells displayed substantial CAR-mediated lysis against primary MM cells in the BM-MNC, but, as expected, there was little or no lysis of CD38^+^ non-MM cells even after using 1000 ng/ml of DOX (Fig 5 and S5 Fig). Furthermore, also as expected, these lower affinity (A4, B1) CAR T cells, showed no autologous T cell killing (fratricide) in contrast to high affinity (028) CAR T cells (S6 Fig).

More interestingly, the cells maintained their lytic activity against MM cells, with no signs of off-tumor effects for longer than 120 hours after the removal of DOX (Fig 5). Taken together these results demonstrated that the DOX inducible CAR design is also a feasible strategy for low-affinity CD38-CAR T cells to establish their therapeutic effects, which could be maintained with no toxic signs for a longer period even after removal of DOX.

## Discussion

In this study we evaluated the feasibility of controlling the on-target, off- tumor effects of CD38-CAR T cells using a DOX inducible Tet-on CAR design. Our results show that this inducible strategy allows the sufficient surface expression of CD38-CARs and thus also the CAR-mediated cytolysis of relevant target cells in a DOX-dose dependent manner. More importantly, we found that the removal of DOX results in a gradual elimination of the off-tumor hematotoxic effects of the high affinity CD38-CAR T cells, especially when DOX exposure dose was limited to 10ng/ml. Somewhat unexpectedly we observed that this approach also generated a small, albeit a temporary therapeutic window after DOX removal, in which the on-tumor effects retained much longer and therefore could be separated from the off-tumor effects. We also collected evidence that this inducible strategy can also be applied to induce the maximal functional expression of our recently developed affinity-optimized CD38-CARs, which already enable T cells to discriminate CD38^high^ MM cells from CD38^int^ normal hematopoietic cells. Collectively, these results indicate the possibility to utilize the DOX inducible CAR-design to actively regulate the CAR-mediated activities of therapeutic T cells. This approach will provide another level of safety to already affinity optimized CD38-CAR T cells, but also increases the risk of tumor immune escape. Therefore, the main benefit of being able to control the high affinity CD38-CAR T cells could be the possibility to use them—as they can recognize lower levels of CD38 expression—in situations where CD38 expression on MM cells is strongly downregulated, such as in the case of post CD38-antibody (e.g. Daratumumab) treatments[34], and as a potential risk when using affinity-optimized CD38-CAR T cells.

From a cell biological point of view, our data collected during the 24 hours induction and subsequent decay phase of high affinity CD38-CARs provide evidence for a linear correlation between the CAR surface expression levels and the cytotoxic activity of CD38-CAR T cells. Related to this, our finding that a certain high affinity CAR expression level results in the separation of the anti-tumor effects of CD38-CAR T cells from their off-tumor cytotoxicity is also novel and suggest that quantitative manipulation of CAR surface expression could, next to CAR affinity optimization, also be exploited to increase the tumor selectivity of CARs directed against tumor-associated antigens (TAAs). Nonetheless, it should be noted that the regulation of gene expression is a highly complex and a difficult to control process. Although we provide evidence that CAR expression in this inducible system is dependent on the dose of DOX, a phase 1 gradual DOX increase may be needed to define a precise dose for each individual to establish a certain level of functional CAR expression. Thus whether the DOX inducible Tet-on strategy will be feasible and the most convenient way for quantitative control of the surface expression of high affinity CD38-CARs needs to be evaluated in future studies. Alternative strategies which are not based on gene regulation may be more feasible for the precise quantitative expression of CARs on the T cell surface, such as the docking of an scFv on an universal receptor[25].

From the safety point of view, our data clearly indicate that the removal of DOX will eventually result in the elimination of off-tumor toxicities of high affinity CD38-CAR T cells. However, one important issue to be discussed is whether the elimination of toxic effects will be sufficiently rapid with this DOX-inducible system. We observed that high affinity CD38-CARs, when they are induced with an optimal dose of 10ng/ml of DOX will lose most, if not all, of their off-target effects within 24 hours (Fig 4). This time frame, when compared to the reported much more rapid clinical results of the suicide gene inducible caspase 9 (iCasp9) system (90% of the cell kill within 30 minutes) is indeed too slow [19]. However, in an *in vitro* system the elimination of iCasp9 positive T cells took also around 24 hours [20]. Moreover, it is known that suicide gene approaches, for instance HSV-TK can cause bystander effects and thereby exhibit safety “leaks”[35,36]. Also, both HSV-TK and the rTta can itself elicit immune response due to their pathogen or foreign origin[37,38]. Furthermore, it needs to be mentioned that it is not precisely known how rapid the elimination of the infused cells should be to prevent further complications. If the direct hematotoxic effects of CD38-CAR T cells needs to be controlled, this time frame does not necessarily be very rapid, since CD38-CAR therapy mainly eliminates NK cells and monocytes, while a large fraction of T and B cells will be ignored as they are CD38 low/negative. The temporary damage to NK cells and monocytes can be gradually recovered after DOX removal since CD38-CARs therapy does not affect CD38 negative normal stem cells [13,14]. It is obvious that DOX removal will also rapidly or eventually abrogate the anti-myeloma effect, depending on the CD38 expression levels on MM cells. But again, the main advantage if this strategy above suicide gene approaches will be that the CAR expression can be re-induced whenever necessary to regain the control on the tumor growth.

In the case of CRS associated with CAR therapy, the immediate disease symptoms can be currently controlled within hours with the use of the anti-IL6 antibody tocilizumab [6,10,39]. Thus, this novel treatment generates a window of opportunity, during which the downregulation of CARs to sufficiently non-toxic levels can be realized. In conclusion, although it may be not as rapid as the suicide gene approach, the inducible CAR strategy can be beneficial, especially when used in combination of tocilizumab in case of CRS. Obviously the main advantage of such an approach will be the control of the therapeutic cells without the need to destroy them permanently.

Another relevant question is whether the Tet-on system is the best or the most convenient system among other inducible systems: when compared to a Tet-off system, a Tet-on inducible system seems more practical to control the toxic effects of CAR T cells since a Tet-on system, where the default is the “off” switch will prevent unfavorable antigen-induced T cell differentiation and exhaustion as compared to a default “on” switch [40]. However, several recently developed strategies such as the CARs with a dimerizer-activated signaling domains [23] may be more effective and rapid than the Tet-on system because they are not dependent on transcriptional gene regulation to achieve the similar effect. Strategies aiming at the induction of CARs in the tumor microenvironment, such as hypoxia inducible CARs[41] or the recently described Syn-Notch strategy[42,43] in which the CAR expression is induced upon recognition of an antigen in the microenvironment also deserve comparison with this more conventional Tet-on strategy. Therefore, while our results are highly promising, we need to acknowledge that an *in vivo* preclinical evaluation of this Tet-on system is necessary to determine whether this strategy can indeed control the CAR T cell related toxicities and the optimal control of DOX half-life and dosing. However, the challenge here is the development of appropriate *in vivo* models where the hematotoxic and other off-tumor effects, as well as the CRS related to CAR T cell therapy, can be adequately mimicked [44].

Nonetheless, the flexibility in CAR functions that we observe in this study illustrates the advantages to control CAR expression and consequently the cytotoxic functions. The development of a controllable switch to effectively tune engineered cells will lead to a safer application of CAR T cell therapy.

## Supporting information

S1 Fig Long-term exposure of MM target cells to TRE-CAR T cells and possibility to re-stimulate TRE-CAR T cells. (A) Lysis of luciferase-transduced CD38^+^ MM cell line UM9 (A) after co-incubation with inducible Mock and high affinity (028) or low affinity (A4 and B1) TRE-CD38-CAR for 6 days with 0 (left panel) or 1000 ng/ml DOX (right panel). Cytotoxicity was measured in flow cytometry-based assay as mentioned in the material and methods. Presented is duplicate measurements +/- SD. (B) BLI-based cytotoxicity assay of 16 hours, after first DOX stimulation (left panel) and after a second DOX stimulation of the same cells, upon culturing without DOX for at least 120 hours (right panel). Presented is a pooled data of two independent experiments, n = 2 +/- SD.
(TIF)

S2 Fig TRE-CAR T cells show a DOX-dependent cytokine release upon incubation with MM-BM. 24 hours after co-incubation with MM-BM, cell supernatants were harvested to measure cytokine secretion (E:T ratio 3:1) with a flow cytometry-based assay. Graph shows the secretion of IFN-γ, TNF and IL-2. Presented is the representative data of cytokine release of five independent experiments.
(TIF)

S3 Fig Different time points for induction of CD38-CAR-induced anti-MM cytotoxicity. Lysis of luciferase-transduced CD38^+^ MM cell line RPMI8226 after co-incubation with inducible Mock and TRE-CD38-CAR, which were treated with (A) no or 1000 ng/ml DOX for 2, 5, 8, 24, 48, 72 and 120 hours or (B) treated with Dox 24 hours and washed and incubated without DOX for 5 or 48 hours. The BLI signal from surviving MM cells was measured after 16 hours using a luminometer and the percentage lysis was calculated as indicated in the material & methods.
(TIF)

S4 Fig Representative flow cytometry density plots of MM-BM. MM-BM samples of patient 1, 2, 3, 4 and 5 were stained for CD38^+^/CD138^+^ expression to illustrate the level of CD38 expression on MM cells (upper right) versus healthy MNCs (upper and lower left).
(TIF)

S5 Fig Off-tumor effect of inducible low affinity CD38-CARA4 T cells. Pooled data obtained from the analysis of five MM patient bone marrow samples (patient 2–5, see for their phenotype data S4 Fig) were co-incubated with inducible low affinity (A4) CD38-CAR T cells (E:T ratio 3:1) treated with DOX according to the schedule Fig 3A. Depicted are the average CAR-dependent lysis of MM cells (CD138^+^/CD38^+^ ; open squares) and lysis of healthy non-MM cells (CD138^-^/CD56^-^/CD38^+/-^ ; grey diamonds) by inducible CD38-CAR T cells. Incubated with DOX for 24 hours 1000 ng/ml (upper left), 48 hours 1000 ng/ml (upper right), 24 hours 10 ng/ml (lower left), 48 hours 10 ng/ml (lower right). Presented is the pooled data of 4 independent experiments mean +/- SEM (2–5, same patients Figs 4 and 5).
(TIF)

S6 Fig Growth rate and cytotoxicity towards autologous Mock T cells. (A) The growth rate of mock and high and low affinity TRE-CAR T cells with 0 (left panel) or 1000 ng/ml DOX (right panel) when cultured on a feeder cell/cytokine mixture. Presented is representative data of five independent experiments. (B) Autologous Mock T cells were labeled and co-incubated with Mock or (high affinity 028 and low affinity A4 and B1) TRE-CAR-T cells with 0 (left panels) or 1000 ng/ml DOX for either 16 hours (upper panels) or 6 days (lower panels) in a flow cytometry-based cytotoxicity assay as described in the material and methods. (C) The level of CD38 expression (mean fluorescent intensity) was measured on the surviving Mock T cells after co-incubation for 16 hours (left panel) or 6 days (right panel) with (high affinity 028 or low affinity A4 and B1) TRE-CD38-CAR T cells in the absence or presence (1000 ng/ml) of DOX.
(TIF)
