# Supplementary figures and images for: Feasibility of controlling CD38-CAR T cell activity with a Tet-on inducible CAR design
Source: PLoS One. 2018 May 30;13(5):e0197349. doi: 10.1371/journal.pone.0197349 (PMC5976165; doi:10.1371/journal.pone.0197349)

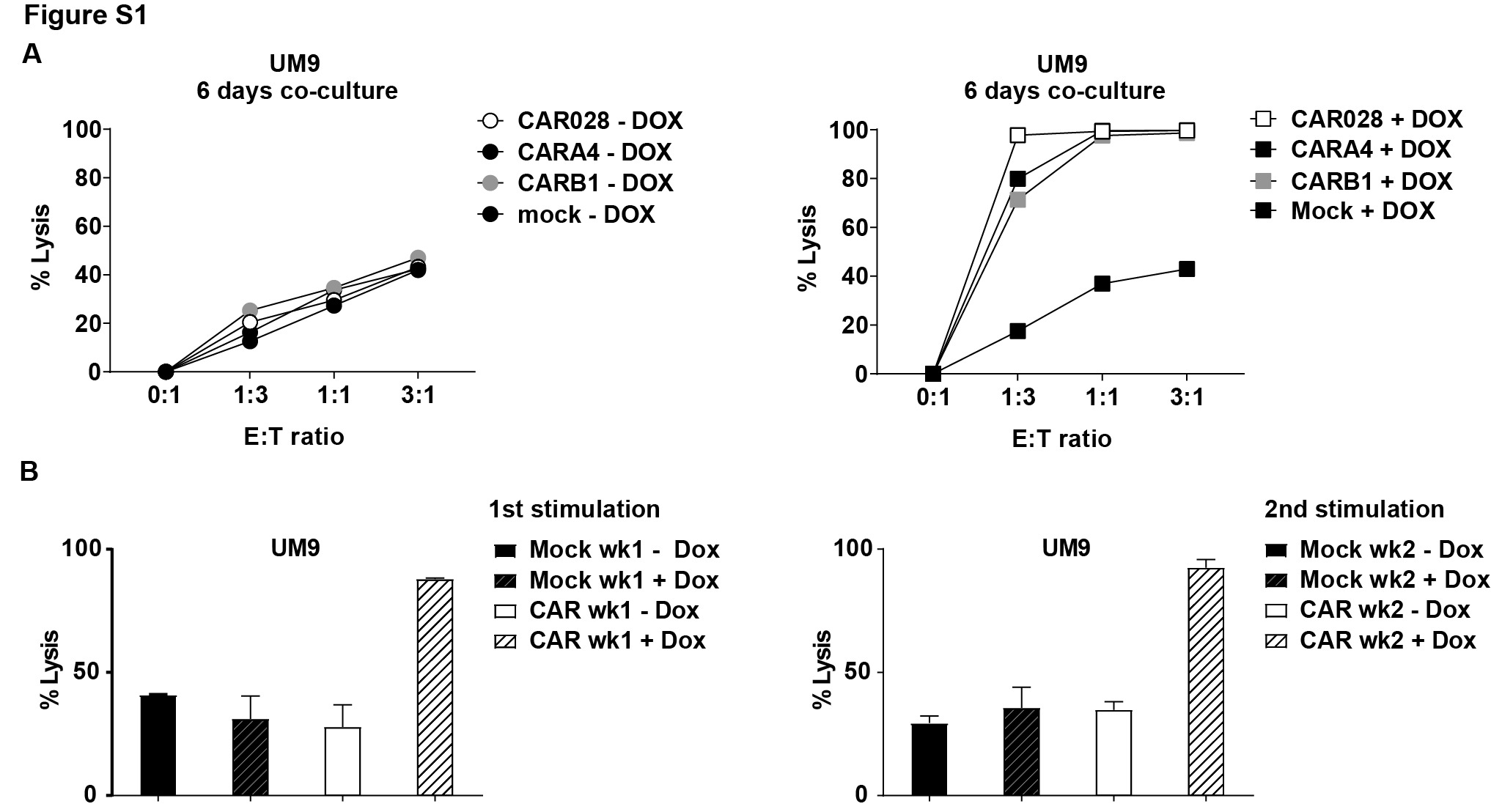

Supplement: S1 Fig — (A) Lysis of luciferase-transduced CD38+ MM cell line UM9 (A) after co-incubation with inducible Mock and high affinity (028) or low affinity (A4 and B1) TRE-CD38-CAR for 6 days with 0 (left panel) or 1000 ng/ml DOX (right panel). Cytotoxicity was measured in flow cytometry-based assay as mentioned in the material and methods. Presented is duplicate measurements +/- SD. (B) BLI-based cytotoxicity assay of 16 hours, after first DOX stimulation (left panel) and after a second DOX stimulation of the same cells, upon culturing without DOX for at least 120 hours (right panel). Presented is a pooled data of two independent experiments, n = 2 +/- SD. (TIF) [file pone.0197349.s001.tif]

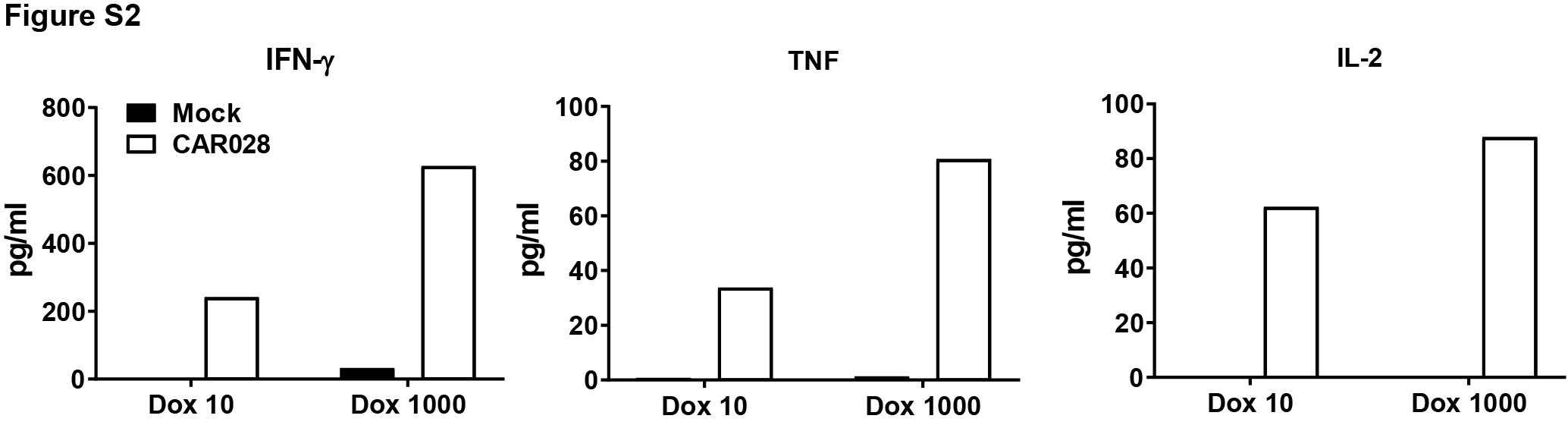

Supplement: S2 Fig — 24 hours after co-incubation with MM-BM, cell supernatants were harvested to measure cytokine secretion (E:T ratio 3:1) with a flow cytometry-based assay. Graph shows the secretion of IFN-γ, TNF and IL-2. Presented is the representative data of cytokine release of five independent experiments. (TIF) [file pone.0197349.s002.tif]

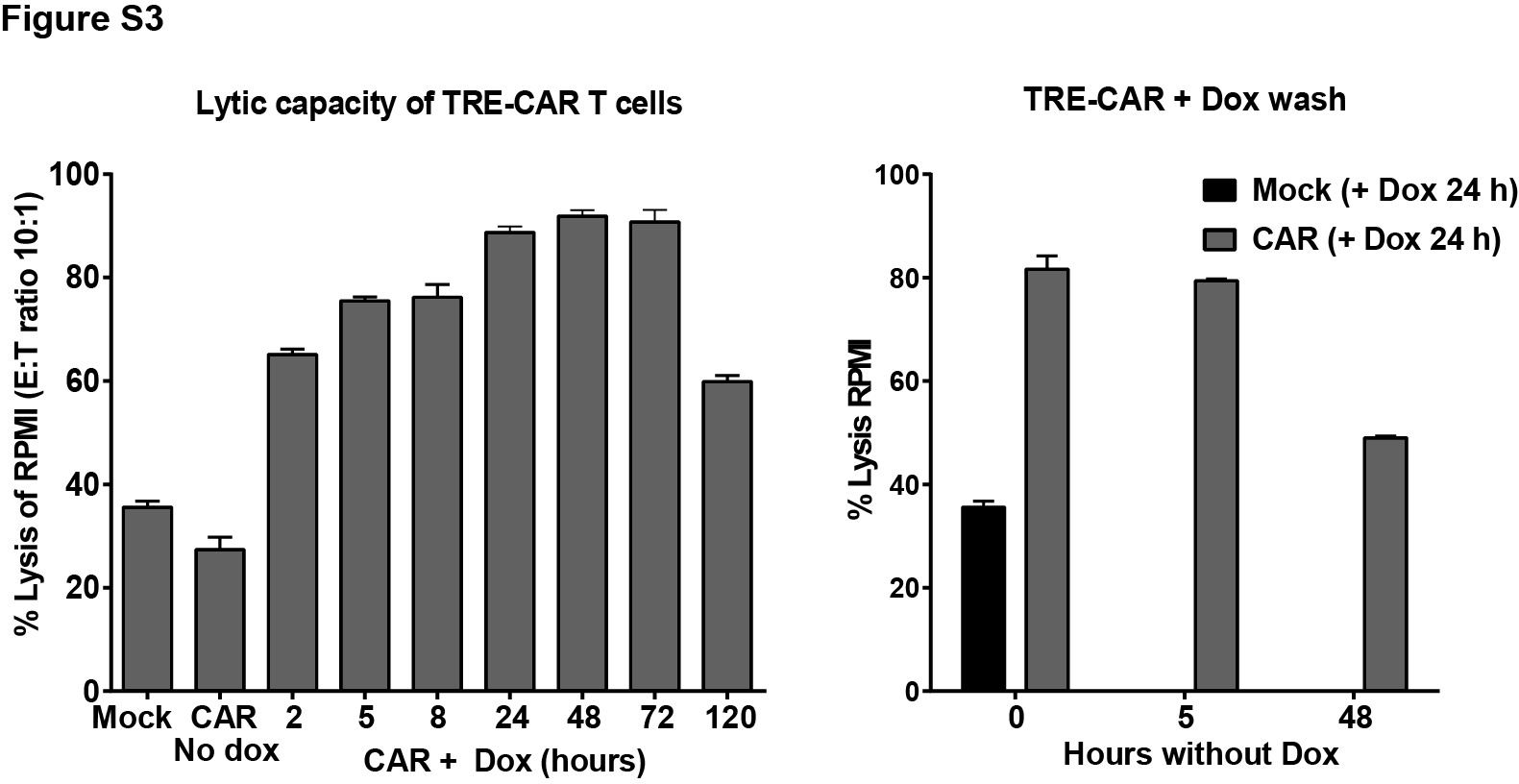

Supplement: S3 Fig — Lysis of luciferase-transduced CD38+ MM cell line RPMI8226 after co-incubation with inducible Mock and TRE-CD38-CAR, which were treated with (A) no or 1000 ng/ml DOX for 2, 5, 8, 24, 48, 72 and 120 hours or (B) treated with Dox 24 hours and washed and incubated without DOX for 5 or 48 hours. The BLI signal from surviving MM cells was measured after 16 hours using a luminometer and the percentage lysis was calculated as indicated in the material & methods. (TIF) [file pone.0197349.s003.tif]

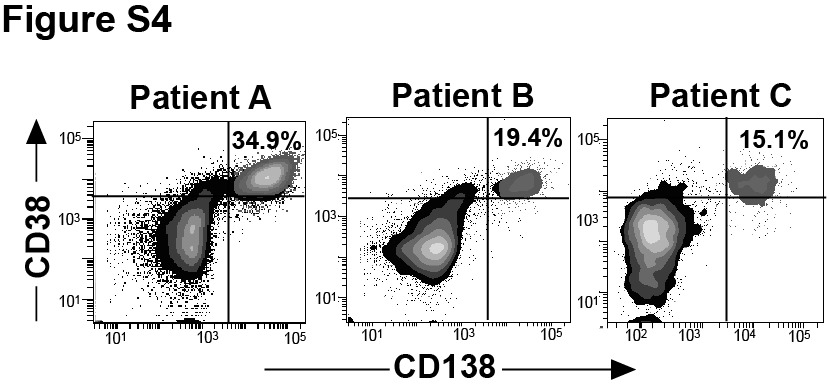

Supplement: S4 Fig — MM-BM samples of patient 1, 2, 3, 4 and 5 were stained for CD38+/CD138+ expression to illustrate the level of CD38 expression on MM cells (upper right) versus healthy MNCs (upper and lower left). (TIF) [file pone.0197349.s004.tif]

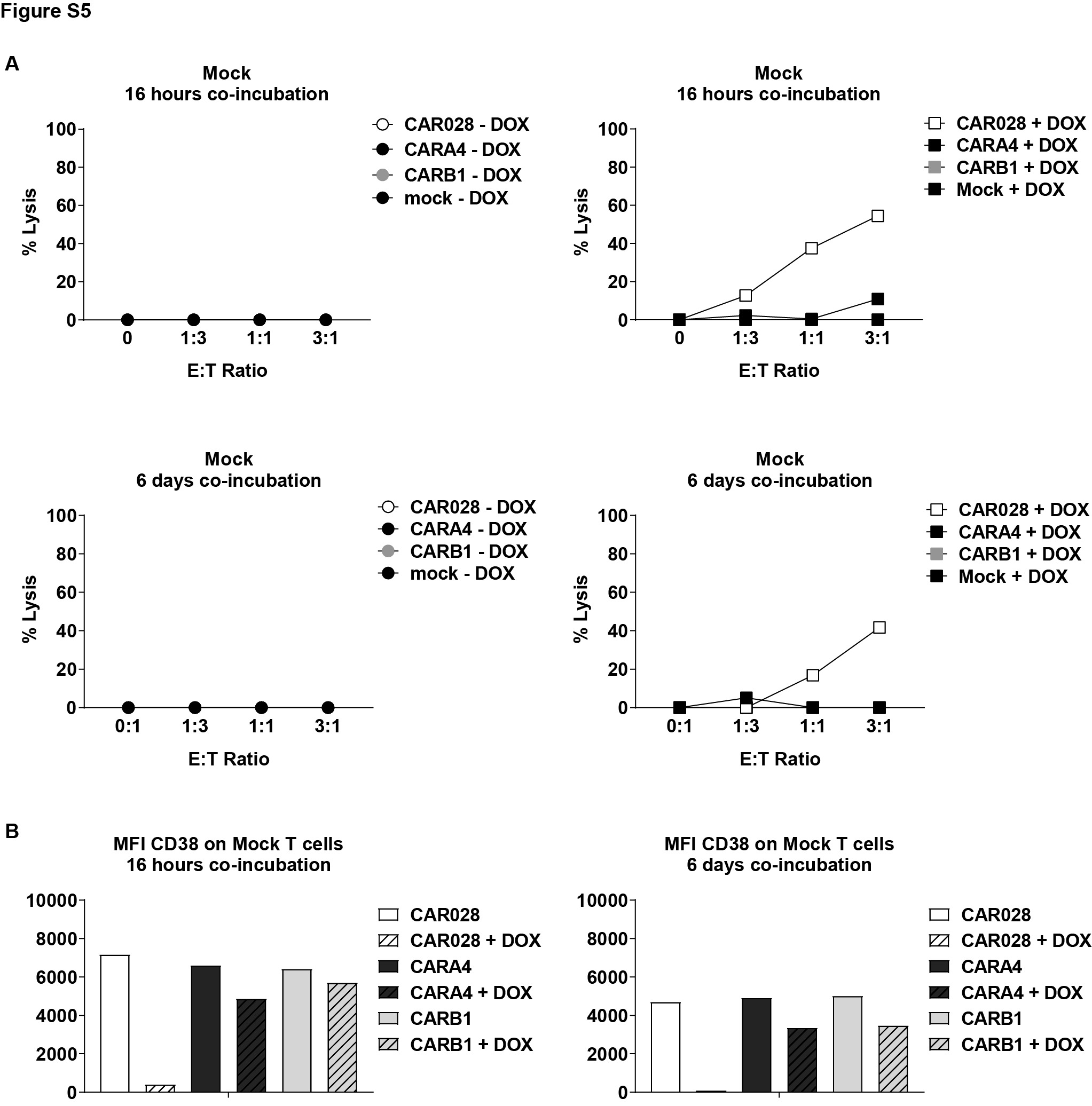

Supplement: S5 Fig — Pooled data obtained from the analysis of five MM patient bone marrow samples (patient 2–5, see for their phenotype data S4 Fig) were co-incubated with inducible low affinity (A4) CD38-CAR T cells (E:T ratio 3:1) treated with DOX according to the schedule Fig 3A. Depicted are the average CAR-dependent lysis of MM cells (CD138+/CD38+ ; open squares) and lysis of healthy non-MM cells (CD138-/CD56-/CD38+/- ; grey diamonds) by inducible CD38-CAR T cells. Incubated with DOX for 24 hours 1000 ng/ml (upper left), 48 hours 1000 ng/ml (upper right), 24 hours 10 ng/ml (lower left), 48 hours 10 ng/ml (lower right). Presented is the pooled data of 4 independent experiments mean +/- SEM (2–5, same patients Figs 4 and 5). (TIF) [file pone.0197349.s005.tif]

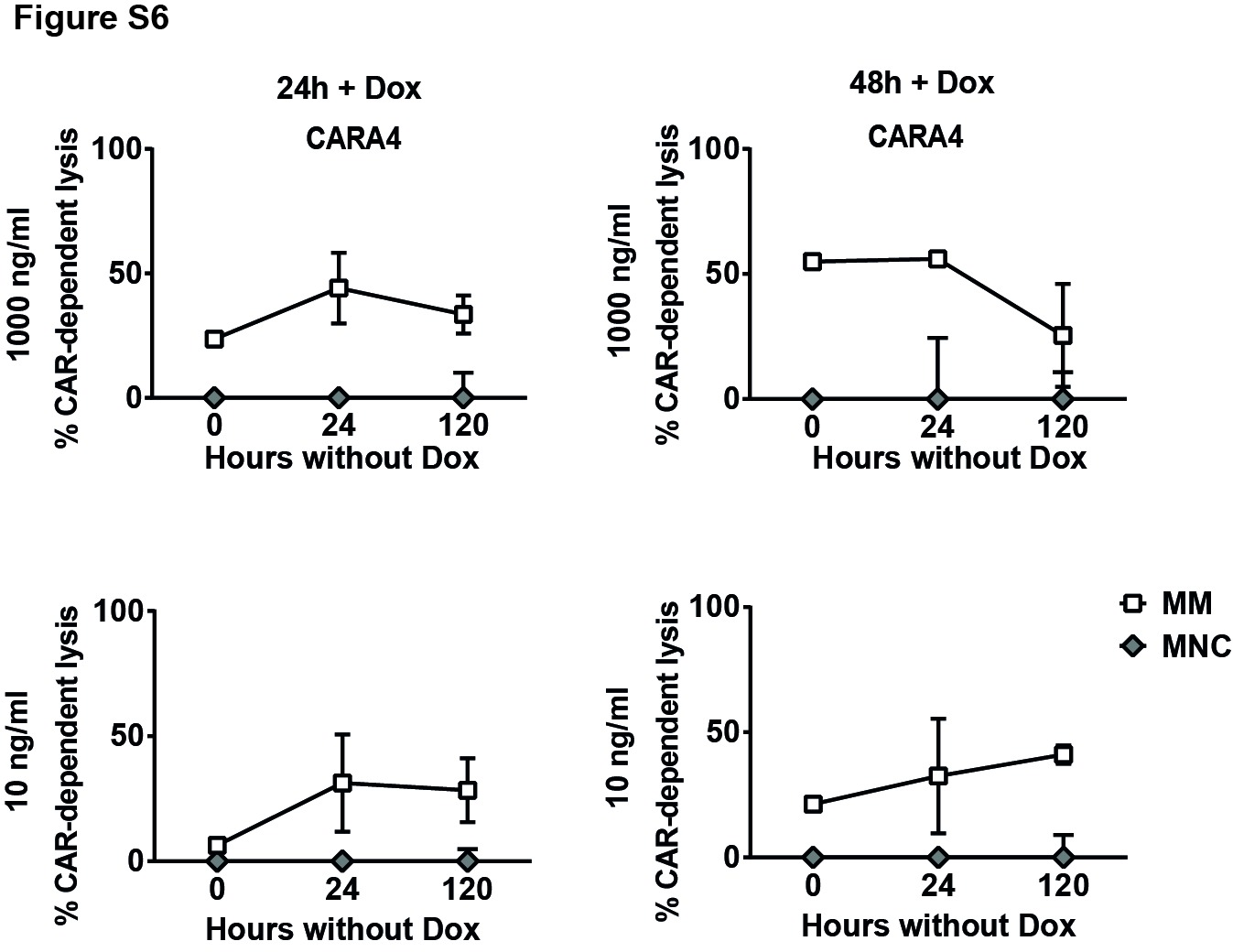

Supplement: S6 Fig — (A) The growth rate of mock and high and low affinity TRE-CAR T cells with 0 (left panel) or 1000 ng/ml DOX (right panel) when cultured on a feeder cell/cytokine mixture. Presented is representative data of five independent experiments. (B) Autologous Mock T cells were labeled and co-incubated with Mock or (high affinity 028 and low affinity A4 and B1) TRE-CAR-T cells with 0 (left panels) or 1000 ng/ml DOX for either 16 hours (upper panels) or 6 days (lower panels) in a flow cytometry-based cytotoxicity assay as described in the material and methods. (C) The level of CD38 expression (mean fluorescent intensity) was measured on the surviving Mock T cells after co-incubation for 16 hours (left panel) or 6 days (right panel) with (high affinity 028 or low affinity A4 and B1) TRE-CD38-CAR T cells in the absence or presence (1000 ng/ml) of DOX. (TIF) [file pone.0197349.s006.tif]
